# Pathological complete response after neoadjuvant chemotherapy for rectal cancer with synchronous multiple liver metastases: a report of an unusual case

**DOI:** 10.1186/s40792-016-0231-9

**Published:** 2016-09-30

**Authors:** Ryosuke Arata, Toshiyuki Itamoto, Satoshi Ikeda, Hideki Nakahara, Akihiko Oshita, Katsunori Shinozaki, Takashi Nishisaka

**Affiliations:** 1 Department of Gastroenterological Surgery, Hiroshima Prefectural Hospital, 1-5-54 Ujina-kanda, Minami-ku, Hiroshima, 734-8530 Japan; 2 Department of Clinical Oncology, Hiroshima Prefectural Hospital, Hiroshima, Japan; 3 Department of Pathology Clinical Laboratory, Hiroshima Prefectural Hospital, Hiroshima, Japan; 4 Department of Gastroenterological and Transplant Surgery, Applied Life Sciences, Institute of Biomedical and Health Sciences, Hiroshima University, Hiroshima, Japan

**Keywords:** Liver metastases, Rectal cancer, Neoadjuvant chemotherapy

## Abstract

**Background:**

Systemic chemotherapy for stage IV colorectal cancer has advanced markedly in the recent years. We report an unusual case of 13 synchronous liver metastases for which a pathological complete response was achieved with neoadjuvant chemotherapy (NAC) consisting of a combination of 5-fluorouracil (5-FU), oxaliplatin, leucovorin (mFOLFOX6), and bevacizumab.

**Case presentation:**

A 44-year-old man was diagnosed with colorectal cancer with synchronous liver metastases. We resected the primary rectal tumor first. Further, after providing NAC for hepatic metastases, lateral segmentectomy and partial resection of the liver were performed. The subsequent result was compatible with a complete pathological response. The postoperative course was uneventful, and the patient is currently alive 5 years after the first surgery without evidence of recurrence and without adjuvant chemotherapy.

**Conclusions:**

For patients with initially resectable colorectal liver metastases, the survival benefits of NAC are still unclear. We report a rare case of 13 synchronous liver metastatic lesions from rectal cancer with a complete pathological response after neoadjuvant bevacizumab-containing chemotherapy.

## Background

Systemic chemotherapy for stage IV colorectal cancer has advanced markedly in recent years following the clinical introduction of two powerful cytotoxic anticancer drugs [1] and molecular-targeted drugs [2, 3]. The tumor response for colorectal liver metastasis (CRLM) has improved with modern combination chemotherapy regimens.

Two distinct strategies for preoperative chemotherapy for CRLM have emerged: neoadjuvant and conversion chemotherapy. Neoadjuvant chemotherapy (NAC) refers to the administration of preoperative chemotherapy for initially resectable CRLM. Although the role of NAC in patients with initially resectable CRLM is still controversial, objectives of this approach include reducing the tumor size, controlling any micrometastases, and avoiding liver surgery in those with rapidly progressive disease.

We report herein an unusual case with 13 liver metastases for which a pathological complete response was achieved with NAC consisting of a combination of 5-fluorouracil (5-FU), oxaliplatin, leucovorin (mFOLFOX6), and bevacizumab, although the patient was considered to have a progressive disease during preoperative chemotherapy.

## Case presentation

**Fig. 1:**
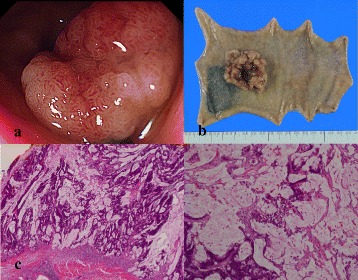
Colonoscopic, macroscopic, and histological findings. **a** Colonoscopy showing a type 2 tumor. **b** Macroscopic findings of the resected rectum. **c** Histological examination of the primary tumor showing mucinous adenocarcinoma

A 44-year-old man was accidentally found to have positive fecal occult blood tests on a regular checkup; therefore, he visited a local hospital for further examination. Colonoscopy showed a type 2 tumor, 34 × 27 mm in size, in the rectum (Fig. 1a). Then, the patient was referred to our hospital for treatment.

Contrast-enhanced computed tomography (CT) showed two hepatic metastases in segments II and IV. We planned to resect the primary rectal tumor first, followed by liver resection. The patient underwent low anterior resection of the rectum, and the resected specimen was a type 2 tumor of the rectum (Fig. 1b). Histological examination of the resected rectum showed mucinous adenocarcinoma (mp, ly2, v0, N1 [1/28]) (Fig. 1c). Contrast-enhanced CT images obtained 1 month postoperatively showed an increase in the number of liver metastases from 2 to 12 lesions. Although it was possible to resect all the metastatic lesions in the liver, we planned to perform hepatectomy following NAC because the disease was rapidly progressive.

**Fig. 2:**
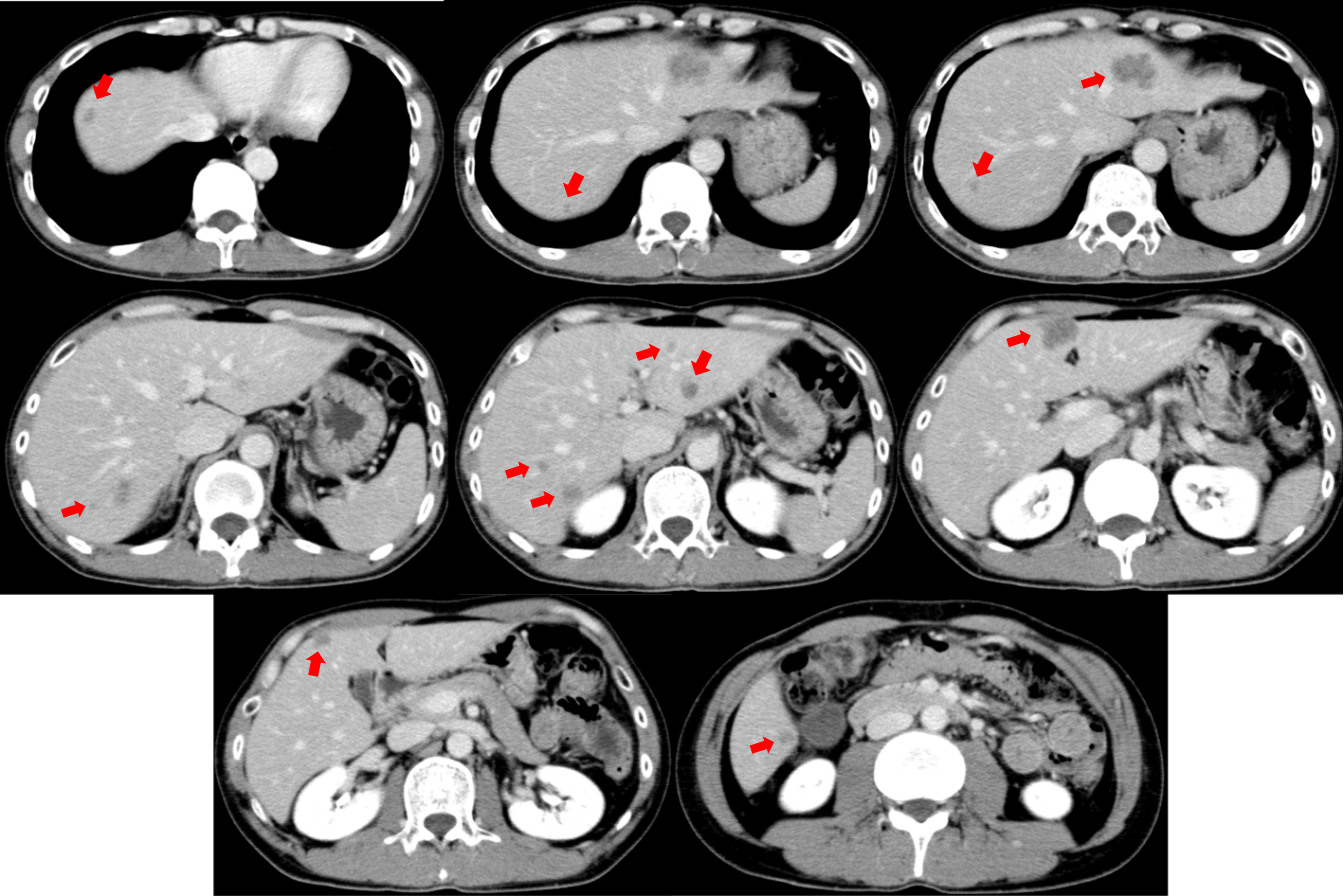
Contrast-enhanced computed tomographic image 1 month after the first operation. The number of liver metastases increased from 2 to 12 lesions

**Fig. 3:**
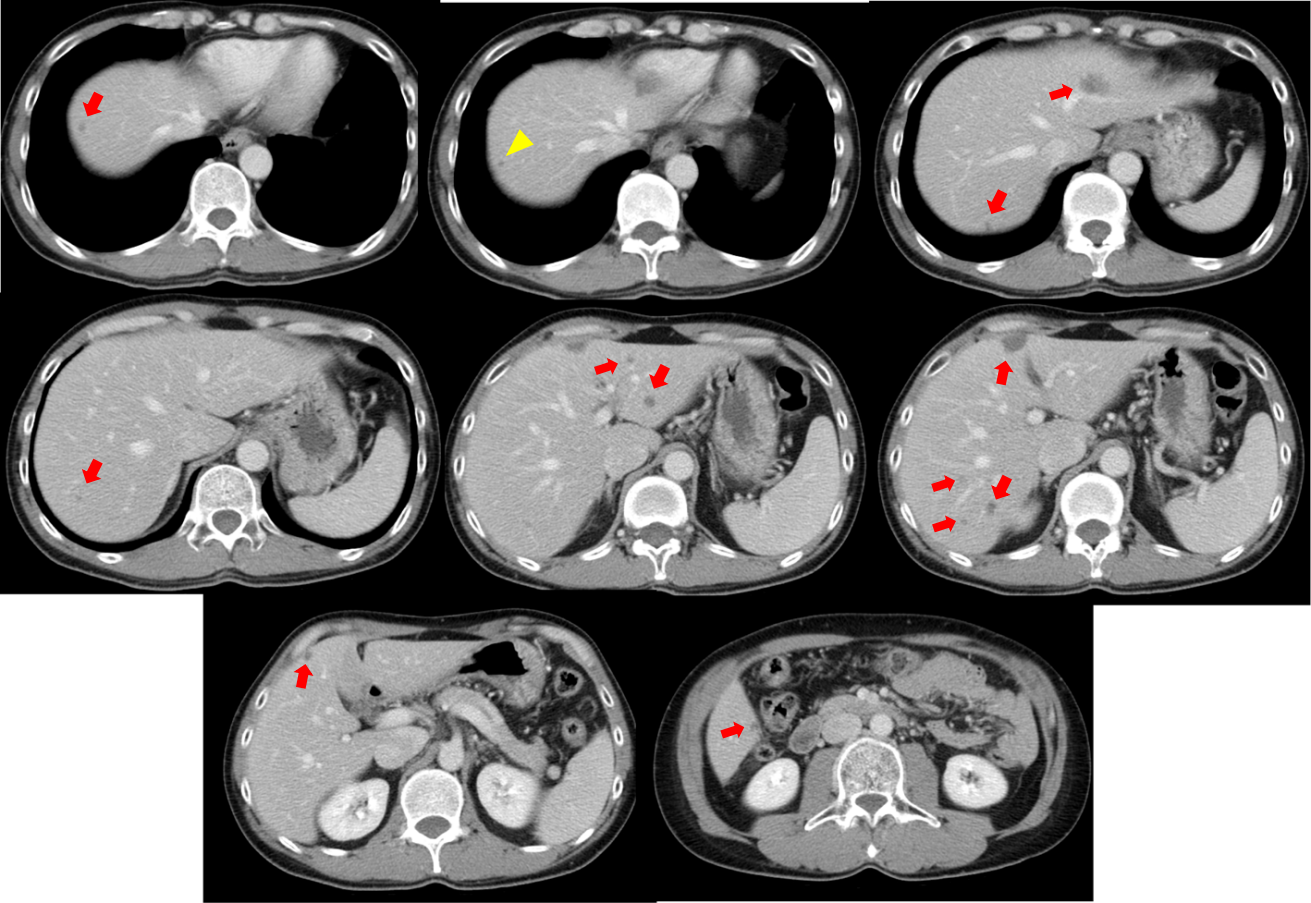
Contrast-enhanced computed tomographic image after 6 cycles of neoadjuvant chemotherapy. The 12 tumors detected before chemotherapy have decreased in size and have had an optimal morphological response. However, a new lesion has emerged in segment VIII of the liver (*arrowhead*)

After 6 cycles of mFOLFOX6 plus bevacizumab, a major response of the liver metastases was documented by using CT. All metastases in the liver decreased in size, and the tumor morphology changed from heterogeneous attenuation, a variable degree of enhancement, and ill-defined borders before treatment to homogeneous, hypoattenuation with well-defined borders (Figs. 2 and 3). However, we evaluated the progression of the disease because the number of metastases increased from 12 before NAC to 13 after 6 cycles of chemotherapy, although the metastases were potentially resectable at this time. Therefore, we added six more cycles of mFOLFOX6 plus bevacizumab followed by chemotherapy consisting of a combination of 5-FU, leucovorin, and bevacizumab. Serum levels of the carcinoembryonic antigen and cancer antigen 19-9 before chemotherapy were 21.7 ng/mL and 746 IU/L, respectively, and they decreased to 2.8 ng/mL and 8 IU/L before hepatectomy, respectively.

He underwent lateral segmentectomy (i.e., resection of segments II and III) and partial resection of segments IV, V, VI, VII, and VIII 1 year after the first operation to confirm that the number of metastases in the liver had not increased further.

**Fig. 4:**
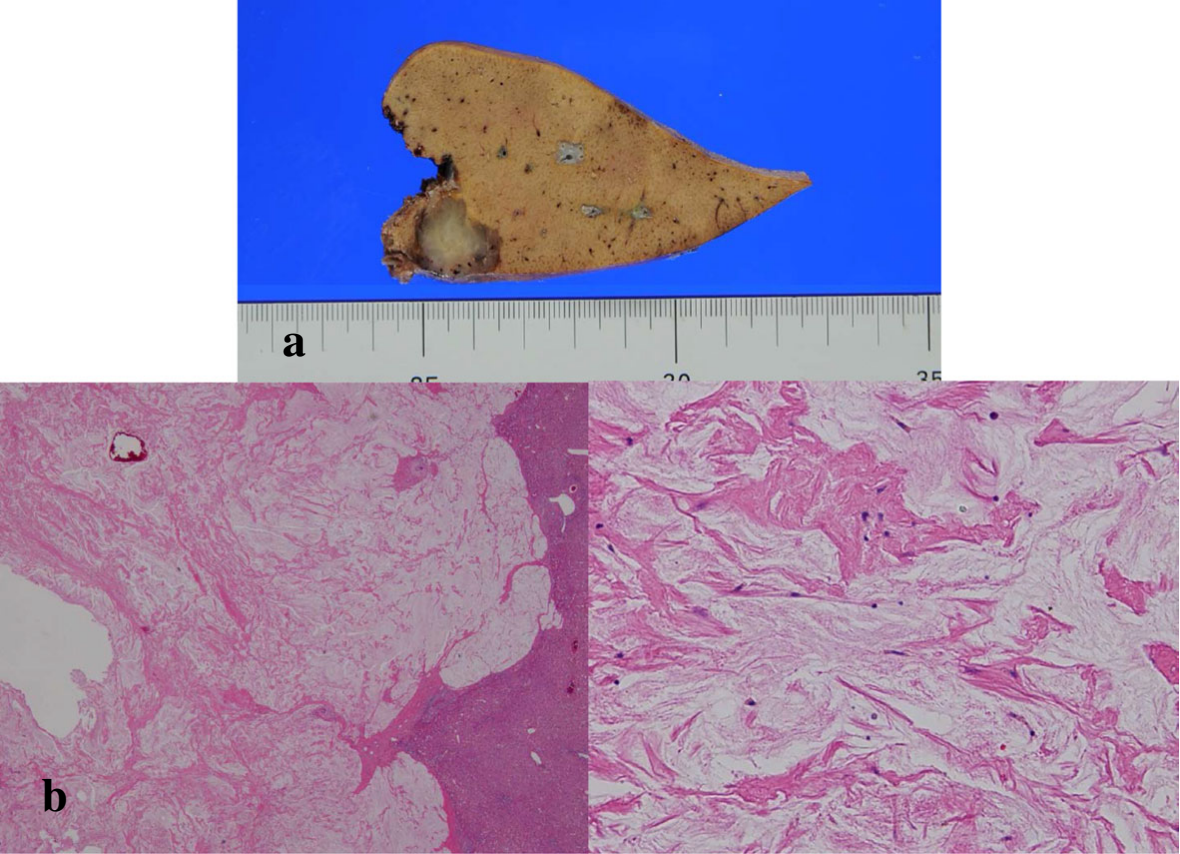
Microscopic and histological findings. **a** Microscopic findings of the resected specimen of the liver (S2). The tumor is white and homogeneous. The boundary between the non-cancerous parts is clear. **b** Histological findings of the tumor show the absence of mucinous adenocarcinoma cells and the presence of only mucous

Macroscopically, the tumor in segment II was white and homogeneous, and it had a clear border (Fig. 4a). Pathological examination demonstrated that all tumors had mucin, fibrotic tissue, and no viable cancer cells (Fig. 4b). These findings were compatible with a complete pathological response. His postoperative course was uneventful, and he is currently alive 5 years after the first surgery without evidence of recurrence despite no adjuvant chemotherapy.

### Discussion

We reported herein a rare case of 13 synchronous liver metastatic lesions from rectal cancer with a complete pathological response after neoadjuvant bevacizumab-containing chemotherapy. Another unusual feature of the case was that the response evaluation on CT after 6 cycles of chemotherapy indicated a progressive disease because a new lesion (the 13th lesion) emerged in the liver.

For patients with initially resectable CRLM, the survival benefit of NAC is still unclear. Potential disadvantages of NAC are the inherent risk of disease progression preoperatively and the risk of liver toxicity. The routine use of NAC for patients with clearly resectable lesions limited to the liver is not recommended owing to a lack of benefit on survival [4].

However, both imaging and pathological responses of liver metastases to preoperative chemotherapy are good prognostic variables [5, 6]. NAC is most beneficial when patients with favorable tumor biology are identified and selected for treatment [6].

A complete radiological response was achieved in 3 to 14 % of patients in the neoadjuvant setting with cytotoxic chemotherapy (oxaliplatin-based or irinotecan-based chemotherapy) for CRLM [7–10]. Even in patients with no metastases observed on imaging, residual macroscopic disease was found in about 25 to 45 % of the patients at the time of operation [11–14]. In patients with no obvious disease at surgery, microscopic cancer was observed in the resected specimen from the site of initial liver metastases in 80 % of the patients. In addition, in patients with no tumors observed and in whom the site of complete response was left in place, in situ recurrence was observed in 74 % of the cases after 1 year [13]. Therefore, a complete response on imaging does not necessarily equate with a complete clinical or pathological response [13, 14]. A pathological complete response was achieved in 2 to 11 % of the patients with only cytotoxic chemotherapy [5, 8, 15].

Bevacizumab is the only anti-angiogenic agent that has been extensively studied in the setting of resectable (or potentially resectable) or unresectable CRLM. Although the complete radiological response rate in patients treated with bevacizumab-containing regimens ranges from 0 to 9 % [16–18], a pathological complete response has been achieved in 8.9 to 16 % of the patients [14, 15, 19]. The discrepancy between radiological and pathological complete response rates may be because of the cytostatic mechanism of bevacizumab. Response evaluation criteria for solid tumors are standard measures used to evaluate the tumor response to treatment; they were developed to assess tumor shrinkage after cytotoxic chemotherapy. However, the criteria may be limited in assessing the response to bevacizumab-containing chemotherapy [19]. Chun et al. demonstrated that CRLMs tend to decrease in size and display unique morphological changes in CT images after bevacizumab-containing chemotherapy. They defined an optimal morphologic response on contrast-enhanced CT images as a change in metastases from lesions with heterogeneous attenuation and thick, irregular borders to homogeneously hypodense masses with a sharp interface between the tumor and adjacent normal liver parenchyma, which in some cases can mimic a cyst [19]. Effective morphological changes correspond to the replacement of tumor cells by fibrosis, pathologically [19].

The optimal timing of primary tumor and liver metastases resection in patients with synchronous resectable CRLM is still controversial. Our strategy has been to perform resection of the primary colorectal tumor first followed by hepatectomy (i.e., staged resection). The patient was treated with NAC for synchronous liver metastases following the resection of primary cancer because the number and size of liver metastases had rapidly increased before the planned hepatectomy.

On contrast-enhanced CT performed after 6 cycles of bevacizumab-containing chemotherapy, all the 12 liver metastases detected before chemotherapy had decreased in size, and radiological findings of the metastases showed effective signs corresponding to the optimal morphologic response [19]. However, we evaluated it as a progressive disease because a new lesion emerged in the liver. The reason why the new lesion, which consequently showed a pathological complete response, was detectable after chemotherapy is unclear. The lesion that was in situ before chemotherapy may have become detectable in CT images owing to its modification to a homogeneous, hypoattenuating lesion with well-defined borders due to the effect of bevacizumab-containing chemotherapy. As a result, the time when the 6 cycles of chemotherapy are completed may be the optimal timing for hepatectomy. As in the present case, it is necessary to evaluate the effectiveness of the treatment carefully when the number of tumors increases during bevacizumab-containing chemotherapy and the optimal morphologic response is identified.

Complete pathologic clearance of all liver metastases after chemotherapy is associated with considerable overall survival, and it is a strong predictor of both prolonged survival and disease cure [5]. Several retrospective studies have demonstrated that in patients with a good pathological response to NAC, adding bevacizumab to the cytotoxic drugs was associated with a better outcome [20–22]. The patient reported herein with more than 10 synchronous liver metastases is alive without recurrence for 4 years since the last operation, and we believe that the disease is cured.

## Conclusions

In conclusion, the survival benefits of NAC are still unclear for patients with initially resectable colorectal liver metastases. We report a rare case of 13 synchronous liver metastatic lesions from rectal cancer with a complete pathological response after neoadjuvant bevacizumab-containing chemotherapy.
